# Measurement properties of pulmonary function measurement and patient-reported outcome measures in patients with multiple rib fracture without flail segment; validation study alongside the multicenter FixCon trial

**DOI:** 10.1007/s00068-026-03338-7

**Published:** 2026-10-09

**Authors:** Maartje L. Luttjeboer, Max R. Van Diepen, Jonne T. H. Prins, Suzanne F. M. Van Wijck, Michael H. J. Verhofstad, Mathieu M. E. Wijffels, Esther M. M. Van Lieshout, FixCon study group, Taco J. Blokhuis, Doeke Boersma, Erik R. De Loos, Elvira R. Flikweert, Frank F. A. IJpma, Sanne Kleinveld, Simon P. Knops, Albert F. Pull ter Gunne, W. Richard Spanjersberg, Gerben Van der Bij, Floortje C. Van Eijck, Pieter J. Van Huijstee, Gust Van Montfort, Jefrey Vermeulen, Dagmar I. Vos

**Affiliations:** Trauma Research Unit Department of Surgery, Erasmus MC, University Medical Center Rotterdam, Rotterdam, The Netherlands

**Keywords:** Multiple rib fractures, PROMs, Quality of life, Reliability, Validity

## Abstract

**Background:**

The reliability and validity of pulmonary function, pain and health related quality of life (HR-QoL) as outcome measures for patients with rib fractures, remain uncertain. Our objective was to evaluate measurement properties in patients with multiple rib fractures by comparing them with generic HR-QoL instruments (EuroQoL-5D-5L [EQ-5D-5L], Short Form-12 [SF-12]).

**Methods:**

A multicenter RCT in adults (*n* = 180) was performed. Spirometry was performed at 30 days, 3, 6, and 12 months after trauma. Patients completed five questions on pain, EQ-5D-5L, SF-12, and two anchor questions on improvement in pulmonary function and thoracic complaints since previous measurement. Reliability, construct validity, responsiveness and floor and ceiling effects were evaluated for all follow-up time-points.

**Results:**

EQ-5D and SF-12 (sub)scales showed adequate internal consistency. Construct and longitudinal validity were adequate for all (sub)scales, except for construct validity of the SF-12 Physical Component Summary. Ceiling effects were present for all pain questions from 30 days onwards, for EQ-5D-5L utility score (US) from 3 months onwards. Responsiveness analysis indicated that spirometry values showed trivial (tidal volume), small (inspiratory capacity, FEV1), or moderate (FVC) degree of change from 30 days to 12 months. All five pain levels, EQ-5D-5L and SF-12 (sub)scales showed moderate to large degree of change form 30 days to 12 months.

**Conclusions:**

Spirometry and pain are valid instruments for monitoring functional recovery in patients after sustaining multiple rib fractures. Responsiveness was confirmed for pain and spirometry (FVC). EQ-5D-5L and SF-12 are valid, reliable, and responsive. Pain and HRQoL are therefore suitable instruments for measuring treatment effects over time.

**Supplementary Information:**

The online version contains supplementary material available at https://doi.org/10.1007/s00068-026-03338-7.

## Highlights

Spirometry and pain are valid instruments for monitoring functional recovery.

EQ-5D-5L and SF-12 are valid, reliable, and responsive

Spirometry (Forced Vital Capacity), pain and HRQoL can be used to measure treatment effects over time

**Supplementary Information:**

The online version contains supplementary material available at https://doi.org/10.1007/s00068-026-03338-7.

## Introduction

Rib fractures have a great impact on patients and society. For patients this is mainly in the context of pain and quality of life [1, 2]. On society, the impact is mainly due to medical expenses and the number of working days lost [3]. Rib fractures occur in 10% of hospitalized patients after trauma and 30% of patients after blunt thoracic trauma [4–6]. The annual costs of rib fractures are estimated to be 54.5 million euro in the Netherlands [3]. The mean direct health care costs for a patient with one or multiple rib fractures were €6,785 [3]. The mean duration of work absence per patient with rib fractures was 44 days [3].

These objective outcomes are clear and widely used, however the patients’ perception on outcome might be even more important. Therefore, Patient Reported Outcome Measures (PROMs) are designed to quantify patients’ subjective perceptions of functional outcome after rib fractures [7]. Patients with rib fractures experience a wide variety of symptoms and disability during their recovery due to the complexity of the injuries [8–10]. Pain, breathlessness, physical disability, and depression or anxiety after surviving major injury are relevant symptom domains [1]. Consequently, rib fracture studies should include outcome measures regarding pulmonary function, pain, and generic health-related quality of life (HRQoL) [9, 11]. However widely used, reliability and validity of these outcome measures for patients with rib fractures are scarcely reported [12].

The aim of this study was to evaluate the measurement properties of spirometry parameters and numeric rating scales (NRS) for pain in patients who sustained multiple rib fractures by comparing them with scores of generic HRQoL instruments (EQ-5D-5L and Short Form-12 [SF-12]). We hypothesized that spirometry, pain and the HRQoL instruments used are all valid, reliable, and responsive instruments for measuring treatment outcome in patients with rib fractures.

## Materials and methods

### Study data

Data were used from all 180 patients who participated in a multicenter randomized controlled trial comparing surgical stabilization of rib fractures (SSRF) and nonoperative treatment in adult patients with multiple rib fractures without a flail segment [13]. This study is registered at the Netherlands Trial Register (NTR7248; registered May 31, 2018). The study was approved by the Medical Research Ethics Committee Erasmus MC (Ref.No. MEC-2018-067 and NL63430.078.17) and Local Ethics Boards of all participating centers.

### Study population

Patients were recruited from January 23, 2019 until June 13, 2023 in 16 hospitals in the Netherlands. Included patients were 18 years or older who presented to the Emergency Department with multiple rib fractures without a flail segment after blunt trauma. Multiple rib fractures were defined as three or more fractured ribs (CT-confirmed) in ribs 4 to 10, without a flail segment defined as three consecutive ribs with two or more fractures in each rib [14]. At least one of the fractures had to be displaced over one shaft-width, or patients needed to have uncontrollable pain defined as at least six points on a Numeric Rating Scale, despite maximum feasible analgesia. Exclusion criteria are listed in detail in the published study protocol [13].

### Pulmonary function and questionnaires

At 30 days, and at three, six, and 12 months after inclusion, pulmonary function was measured using a handheld spirometer (MicroLoop, Carefusion, PT Medical BV, Leek, The Netherlands). The parameters tidal volume (TV), forced vital capacity (FVC), inspiratory capacity (IC_lung_), and volume during the first second of forced expiration (FEV_1_) were determined. At the same days and two weeks after inclusion, patients were asked to complete the EQ-5D-5L and SF-12 questionnaires and five questions to report pain levels at rest, at night, and during maximum inspiration, self-care, and activities of daily living [13]. At this two-week time point, patients were also asked to complete these questionnaires for their pre-trauma situation.

The EQ-5D-5L is a validated instrument for measuring HRQoL [15, 16]. The EQ-5D utility score (EQ-US) ranges from 0 to 1 and is determined from five 1-item domains: mobility, self-care, usual activities, pain/discomfort, and anxiety/depression. Each item has five possible answers. In addition, the individual’s rating of his/her quality of life state is recorded using a standard Visual Analog Scale (EQ-VAS) ranging from 0 to 100. Higher scores represent better HRQoL. SF-12 is a validated instrument that analyzes global health status. The score is calculated based on 12 items that represent eight domains and are summarized into a Physical Component Summary (PCS) and a Mental Component Summary (MCS). Data from the US population of 1998 were used for transforming all domains to a mean of 50 points and a standard deviation of 10 points [17]. The pain questions were single item questions scored on a Numeric Rating Scale ranging from zero (no pain) to 10 (highest pain level imaginable).

Baseline characteristics collected were age, sex, American Society of Anesthesiologists (ASA) classification, specific comorbidities (i.e., asthma, chronic obstructive pulmonary disease (COPD), or psychiatric/psychological disorders), and smoking status. Injury details collected were the number of fractured ribs and rib fractures (as each rib can have multiple fractures), thoracic injury severity (i.e., Maximum Abbreviated Injury Score, MAIS thorax), and overall injury severity (i.e., Injury Severity Score, ISS).

### Statistical analysis

Data were analyzed using the Statistical Package for the Social Sciences (SPSS) version 28 (IBM Corp. Released 2021. IBM SPSS Statistics for Windows, Version 28.0. Armonk, NY: IBM Corp). The Receiver Operating Characteristic (ROC) curve and Youden Index were analyzed using MedCalc (MedCalc Statistical Software version 18.2.1 (MedCalc Software bvba, Ostend, Belgium; 2018). Missing data were not imputed. Measurement properties of spirometry outcomes and questionnaire (sub)scales were determined in compliance with the COnsensus-based Standards for the selection of health Measurement Instruments (COSMIN) guidelines [18]. Data are reported following the STrengthening the Reporting of OBservational studies in Epidemiology (STROBE) [19].

Descriptive statistics were used for describing the study population. Measurement properties of the spirometry outcomes and pain (sub)scales were determined by comparing them with general health-related quality of life instruments EQ-5D-5L and SF-12.

Reliability was assessed by evaluating internal consistency. Reliability is defined as the extent to which items can be distinguished from each other, despite measurement errors [20]. Internal consistency is defined as the extent to which items in a (sub)scale are intercorrelated, thus measuring the same construct [20]. Inter-item correlations within a (sub)scale were calculated using Cronbach’s alpha. Internal consistency is considered sufficient if the Cronbach’s alpha value is between 0.70 and 0.95, conditional on the scale being unidimensional and a sample size of 10 per item in the instrument, with a minimum of 100 patients [20]. The data for the follow-up moments from pre-trauma to 12 months were used.

Validity is the degree to which a questionnaire measures the construct it is supposed to measure [20]. With no gold standard available, validity of the instruments in this study was expressed in terms of the construct validity, which is defined as the extent to which scores on a specific questionnaire relate to other measures in a way that is in agreement with prior theoretically derived hypotheses concerning the concepts that are being measured [20]. The 30 days data were used to determine the construct validity, as the heterogeneity in recovery (and hence variety in score) was expected to be the most at 30 days. Since the Shapiro-Wilk test showed all (sub)scales were not distributed normally, Spearman rank correlations between the spirometry outcomes and (sub)scales of the pain questions, EQ-5D-5L, and SF-12 were determined to evaluate construct validity. Strengths of correlations were categorized as high (correlation coefficient *r* > 0.6), moderate (0.3 < *r* < 0.6), or low (*r* < 0.3) [21]. Validity was defined as discriminative if the correlation was < 0.3 and as convergent if the correlation was > 0.6. Construct validity was scored sufficient if at least 75% of the results were in accordance with prior predicted hypotheses in a (sub)sample of at least 50 patients [20]. Two authors (MLL and EMMVL; Supplemental Table 1 A) agreed on the predicted correlations between the (sub)scale scores.

Responsiveness is defined as the ability of a questionnaire to detect clinically important changes over time [22]. Longitudinal validity and Effect Size (ES) were evaluated as a measure of responsiveness. Longitudinal validity refers to the extent to which change in one measurement instrument relates to a corresponding change in a reference measure [23]. Longitudinal validity was assessed by testing predefined hypotheses about the expected correlations between changes in spirometry outcomes and (sub)scale scores of pain, EQ-5D-5L, and SF-12. Change scores were calculated as the difference in score between the 30 days and 12 months follow-up measurements. Strengths of correlation were categorized as high (*r* > 0.6), moderate (0.3 < *r* < 0.6), or low (*r* < 0.3) [21]. Since all change scores were not distributed normally based on the Shapiro-Wilk test, Spearman correlation coefficients were calculated. Longitudinal validity was considered sufficient if at least 75% of the results were in accordance with predefined hypotheses in a (sub)sample of at least 50 patients [20]. The correlations between the (sub)scale scores were predicted in consensus between two authors (MLL and EMMVL; Supplemental Table 1B).

The Effect Size (ES) was calculated as measure of the magnitude of change over time. ES was calculated as the mean change in score between two time points (i.e., score at 12 months minus score at 30 days) divided by the standard deviation of the first measurement [24]. An ES < 0.2 is considered trivial, 0.2 to < 0.50 small, 0.5 to < 0.8 moderate, and ≥ 0.8 large magnitude of change. Moderate to large ES was expected; at six months functional limitations were expected, and at 12 months full recovery (or at least much improvement) was expected for most patients.

Floor and ceiling effects are defined as the number of respondents who achieved the lowest or highest possible score, respectively [20]. These effects are present if more than 15% of the study population rates the worst or best possible score on any questionnaire (sub)scale [25]. If floor or ceiling effects are present, extreme items might be missing from the upper or lower ends of the scale, and could reduce content validity. Also, patients with the worst or best possible scores cannot be distinguished from one another, limiting reliability [20]. Floor and ceiling effect were determined for each follow-up moment separately.

The Minimal Important Change (MIC) is defined as the smallest measurable change in outcome score that patients perceive as significant [26]. An anchor-based method was used, as this also indicates importance of the observed change to the patient [20]. In addition to the spirometry and above mentioned questionnaires, patients were asked to complete two anchor question at 30 days and at three, six, and 12 months. These anchor questions evaluated their perception of change in pulmonary function and general condition of the thorax, respectively. The questions were: ‘How would you judge your pulmonary function at this point, compared with your prior follow-up visit?’ and ‘How would you judge the complaints of your chest at this point, compared with your prior follow-up visit?’ The items scored from 1 ‘much better’ through 2 ‘slightly better’, 3 ‘no change’, 4 ‘slightly worse’, or 5 ‘much worse’. The anchor question is only adequate if a Spearman’s rank correlation between the anchor and the change score was > 0.29 [27]. The corresponding change score (i.e., score at time of completion of the anchor question minus the score at the prior follow-up moment) of patients who answered the anchor question as ‘slightly better’ can be considered the MIC [27].

The Smallest Detectable Change (SDC) is defined as the smallest intra-personal change in score that represents (with *p* < 0.05) a ‘real’ difference above the measurement error [28]. To ascertain that patients were stable in the interim period, SDC was based on the change scores of patients who answered ‘no change’ on the transition item. Standard Error of the mean (SEM) can be considered a measure of absolute measurement error [20].

## Results

### Patient and injury characteristics

A total of 180 patients were included, 90 were randomized to SSRF and 90 to nonoperative treatment. The median age was 63 years (P_25_-P_75_ 53–75) and 128 (71%) were males. Eighty-five patients (57%) had an ASA score of 2. Pulmonary comorbidities were present as follows: 13 (7%) patients had asthma, 15 (8%) COPD, 32 (18%) were current smokers and 71 (39%) patients had a history of smoking. The MAIS thorax score was 3 in 162 (90%) patients and the median ISS was 13 (P_25_-P_75_ 9–16). The patients had a median of 6 (P_25_-P_75_ 5–8) fractured ribs and a median of 8 (P_25_-P_75_ 6–10) rib fractures.

### Changes in spirometry and PROM (sub)scales over time

Box-whisker plots of pulmonary function showed that FVC and FEV1 continue to improve over time, while IC and TV show minor to no improvement (Supplemental Fig. 1). All pain items improved over time (Supplemental Fig. 2). The SF-12 MCS subscales remained unchanged and stayed within the population-standardized values, PCS subscales remained below the population norm and some improved over time but did not return to pre-trauma levels (Supplemental Fig. 3). Regarding EQ-US and VAS both improved until approximately six months post-trauma.

### Reliability

Internal consistency was adequate for all (sub)scales of the EQ-5D-5L and SF-12 with a Cronbach’s alpha between 0.70 and 0.95, except for the general Mental Health (MH) and Physical Functioning (PF) subscales of the SF-12 after 30 days (0.530 and 0.687; Table 1). The outcome of the EQ-5D-5L should be interpreted carefully, since the total scale is not unidimensional. Spirometry results, all pain scales, EQ-5D-5L VAS, and four SF-12 subscales are not shown because internal consistency (which is an inter-item correlation) does not apply to a single-item domain.

**Table 1:** Internal consistency (Cronbach’s alpha) of the HRQoL instruments in patients with multiple rib fractures

		Nr. of items	Pre-trauma (*N* = 175)	14 days (*N* = 174)	30 days (*N* = 172)	3 months (*N* = 169)	6 months (*N* = 167)	12 months (*N* = 164)
EQ-5D-5L		5	0.737^a^	**0.694** ^**a**^	**0.699** ^**a**^	0.803^a^	0.790^a^	0.807^a^
SF-12	PF	2	0.762	**0.604**	**0.530**	0.758	0.786	0.801
	RP	2	0.914	0.843	0.872	0.889	0.911	0.948
	RE	2	0.843	0.884	0.807	0.885	0.903	0.928
	MH	2	**0.688**	0.721	**0.687**	0.763	0.706	0.775
	PCS	4	0.852	0.745	0.787	0.875	0.892	0.905
	MCS	4	0.834	0.727	0.782	0.872	0.862	0.894

Data are shown for all follow-up moments. The maximum number of patients was 180, minimal response rate was 164 (91.1%). Bold underlined values indicate they are without the adequacy limits for Cronbach’s alpha (0.70 to 0.95)

^a^ Value should be interpreted carefully, since the total scale is not unidimensional

Spirometry results, all pain scales (inspiration, rest, night, self-care, and ADL), EQ-5D-5L VAS, and four SF-12 subscales (BP, GH, VT, and SF) consist of a single item. They are not shown because internal consistency does not apply to a single-item domain

ADL, Activities of Daily Life; BP, Bodily Pain; EQ-5D, EuroQoL-5D Assessment with 5 Level response options; GH, General Health perceptions; mo, months; MCS, Mental Component Summary; MH, general Mental Health; PCS, Physical Component Summary; PF, Physical Functioning; QoL, Quality of Life; RE, Role limitations due to Emotional problems; RP, Role limitations due to Physical health; SF, Social Functioning; SF-12, Short Form-12; US, Utility Score; VAS, Visual Analog Scale; VT, Vitality, energy, or fatigue; we, weeks

### Construct validity

Table 2 shows a correlation plot across (sub)scales at 30 days follow-up, with 18 calculated correlations for each (sub)scale. The predicted correlations are shown in Supplemental Table 1 A. The number of correctly predicted correlations varied from 13 (72%; SF-12 PCS) to 18 (100%; pain during inspiration and at night, EQ-VAS and SF-12 MCS). Construct validity was adequate for all (sub)scales with > 75% of the 18 correct predicted correlations, except for the SF-12 PCS with 13 (72%) hypotheses predicted correctly.

**Table 2:**
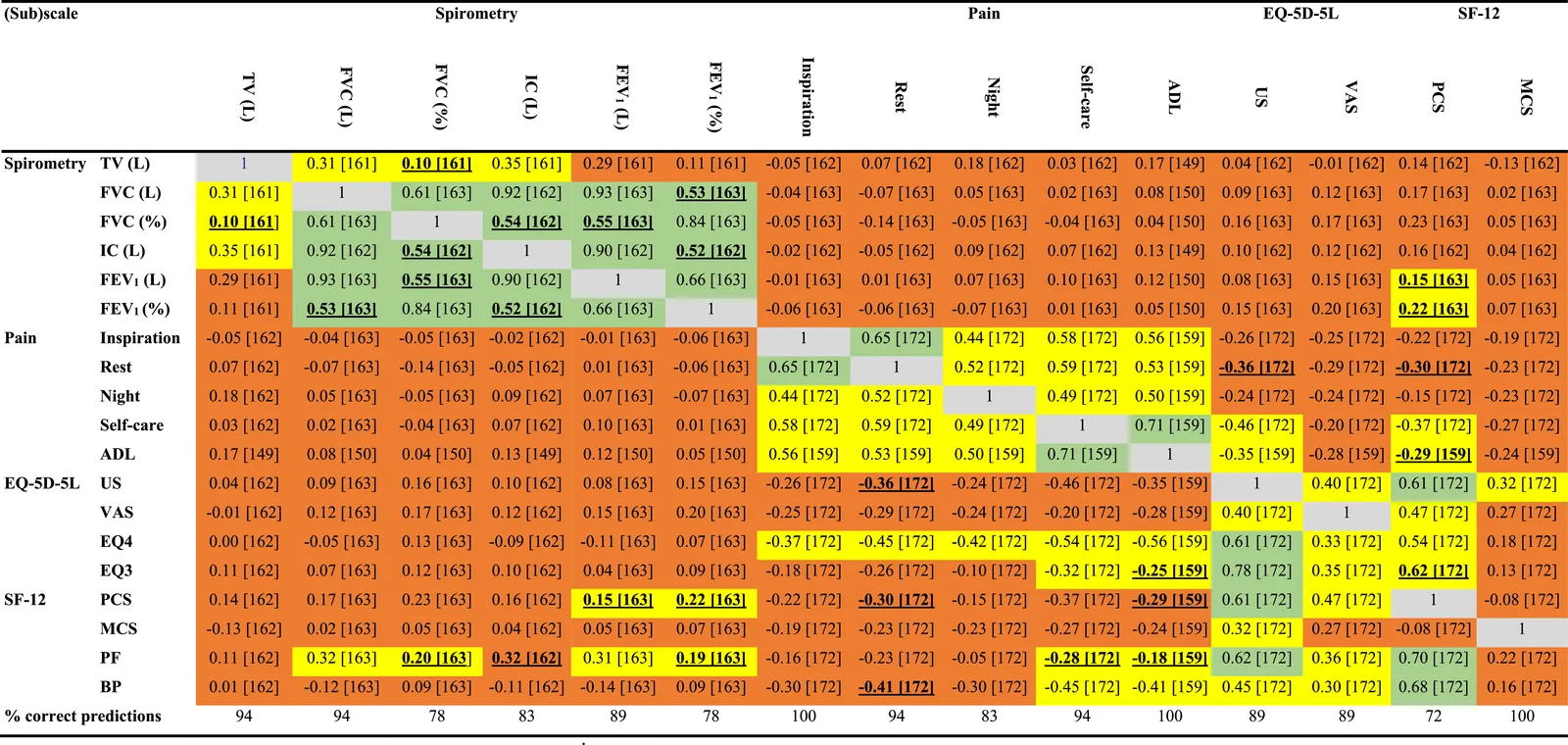
Construct validity of the instruments in patients with multiple rib fractures

Data are shown for the 30 days follow-up. The maximum number of patients was 180, minimal response rate was 159 (88.3%). Construct validity is shown as Spearman’s rank correlation coefficients (*r*) with the number of patients included in the correlation between square brackets. *r* > 0.6 represents high correlation, 0.3 < *r* < 0.6 moderate correlation, and *r* < 0.3 low correlation. Bold and underlined correlations were not predicted correctly. The colors represent low (orange), moderate (yellow) and high (green) predicted correlations

ADL, Activities of Daily Life; BP, Bodily Pain; EQ-5D, EuroQoL-5D Assessment with 5 Level response options; FEV1, Forced Expiratory Volume in 1 s; FVC, Forced Vital Capacity; IC, Inspiratory Capacity; L, Liter; MCS, Mental Component Summary; PCS, Physical Component Summary; PF, Physical Functioning; QoL, Quality of Life; SF-12, Short Form-12; TV, Tidal Volume; US, Utility Score; VAS, Visual Analog Scale

### Responsiveness

Table 3 shows a correlation plot of the change scores, with 18 calculated correlations for each (sub)scale. The predicted correlations are shown in Supplemental Table 1B. The number of correctly predicted correlations varied from 14 (78%; pain during self-care, EQ-VAS, EQ-4) and 18 (100%; TV, FVC, FEV1, pain during inspiration and at rest). Longitudinal validity was adequate for all (sub)scales with 78% to 100% of the hypotheses predicted correctly, where > 75% is required (Table 3).

**Table 3:**
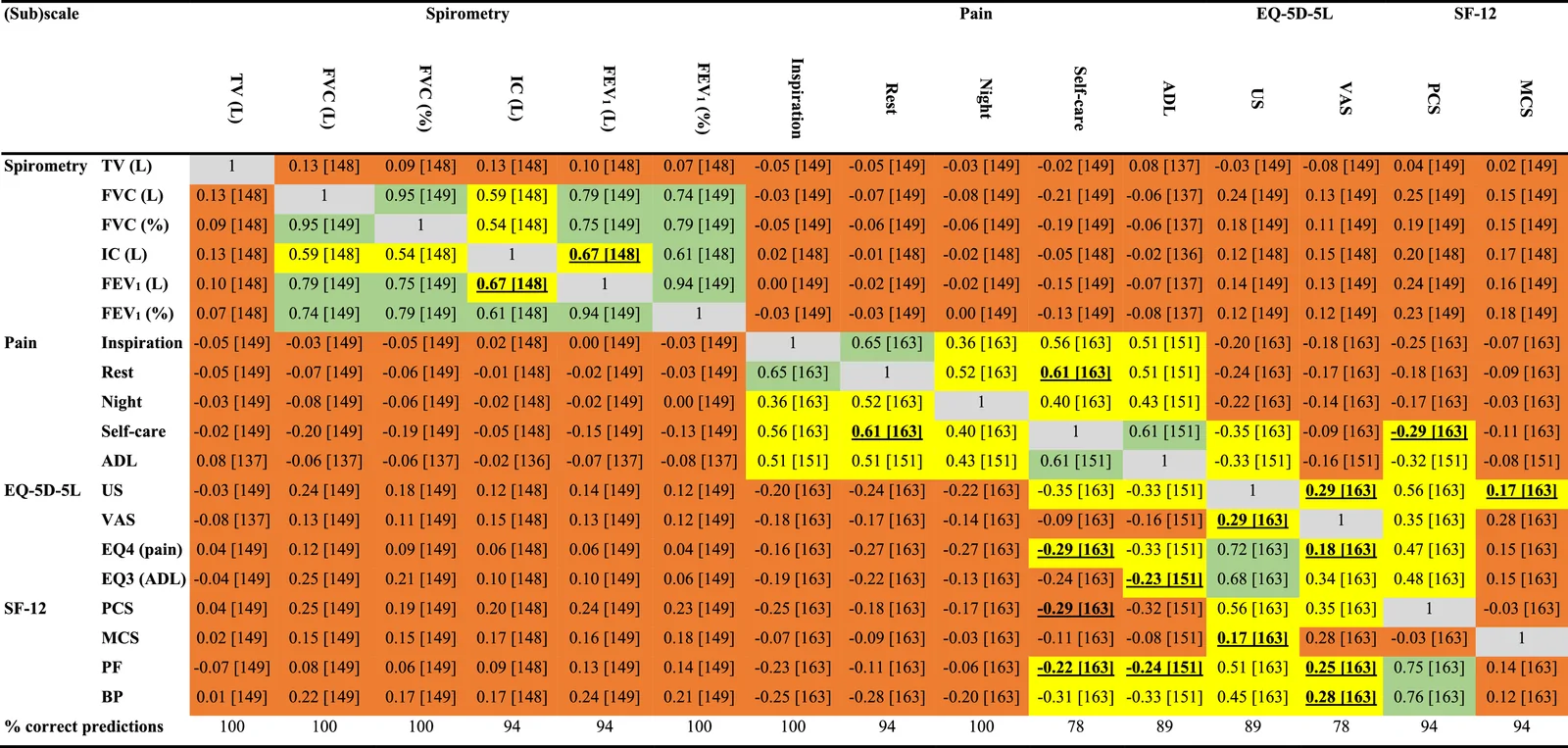
Longitudinal validity of the instruments in patients with multiple rib fractures

Responsiveness is shown as Spearman’s rank correlation coefficients (*r*) of change in scores between 30 days and 12 months with the number of patients included in the correlation between square brackets. The maximum number of patients was 180, the minimum response rate was 136 (75.6%)

*r* > 0.6 indicates high correlation, 0.3 < *r* < 0.6 moderate correlation, and *r* < 0.3 low correlation. Bold and underlined correlations were not predicted correctly. The colors represent low (orange), moderate (yellow) and high (green) predicted correlations

ADL, Activities of Daily Life; BP, Bodily Pain; EQ-5D, EuroQoL-5D Assessment with 5 Level response options; FEV1, Forced Expiratory Volume in 1 s; FVC, Forced Vital Capacity; IC, Inspiratory Capacity; L, Liter; MCS, Mental Component Summary; PCS, Physical Component Summary; PF, Physical Functioning; QoL, Quality of Life; SF-12, Short Form-12; TV, Tidal Volume; US, Utility Score; VAS, Visual Analog Scale

The effect size indicated that in the spirometry parameters a moderate magnitude of change was shown for FVC (%), all other (sub)scales showed trivial to small change (Table 4). All five pain levels showed a moderate to large magnitude of change from 30 days to 12 months. The EQ-5D-5L showed a moderate to large change for all (sub) scales, except for a small magnitude of change for the EQ- pain domain. The SF-12 (sub)scales showed moderate to large magnitude of change from 30 days to 12 months, except for a small magnitude of change for the RE, MH and MCS subscales.

**Table 4:** Responsiveness: Effect Size (ES) of the instruments in patients with multiple rib fractures

(Sub)scale		*N*	Mean change	SD_30 days_	ES	Magnitude of change
Spirometry	TV (L)	149	-0.01	0.57	-0.02	**trivial**
	FVC (L)	149	0.47	1.04	0.45	**small**
	FVC (%)	149	10.60	18.15	0.58	moderate
	IC (L)	148	0.43	0.97	0.44	**small**
	FEV_1_ (L)	149	0.29	0.90	0.32	**small**
	FEV_1_ (%)	149	8.81	19.81	0.44	**small**
Pain	Inspiration	163	-1.72	2.07	-0.83	large
	Rest	163	-0.83	1.58	-0.52	moderate
	Night	163	-1.42	2.21	-0.64	moderate
	Self-care	163	-1.56	1.79	-0.87	large
	ADL	151	-1.39	1.75	-0.80	large
EQ-5D-5L	US	163	0.12	0.18	0.68	moderate
	VAS	163	13.50	15.83	0.85	large
	Pain (Q4)	163	0.03	0.07	0.45	**small**
	ADL (Q3)	163	0.05	0.06	0.82	large
SF-12	PCS	163	11.11	8.82	1.26	large
	MCS	163	2.70	9.96	0.27	**small**
	PF	163	8.85	9.31	0.95	large
	RP	163	9.72	7.95	1.22	large
	BP	163	11.13	11.16	1.00	large
	GH	163	6.16	10.05	0.61	moderate
	VT	163	7.59	9.70	0.78	moderate
	SF	163	9.23	12.77	0.72	moderate
	RE	163	2.33	10.60	0.22	**small**
	MH	163	3.78	9.65	0.39	**small**

Change scores were calculated from 30 days to 12 months. The maximum number of patients was 180, the minimum response rate was 148 (82.2%). Bold underlined text indicates that the magnitude of changes was less than expected (moderate to large)

ADL, Activities of Daily Life; BP, Bodily Pain; EQ-5D, EuroQoL-5D Assessment with 5 Level response options; ES, Effect Size; FEV1, Forced Expiratory Volume in 1 s; FVC, Forced Vital Capacity; GH, General Health perceptions; IC, Inspiratory Capacity; L, Liter; MCS, Mental Component Summary; MH, general Mental Health; PCS, Physical Component Summary; PF, Physical Functioning; QoL, Quality of Life; RE, Role limitations due to Emotional problems; RP, Role limitations due to Physical health; SD_30days_, Standard Deviation at 30 day follow-up; SD_change_, Standard Deviation of mean change; SF, Social Functioning; SF-12, Short Form-12; TV, Tidal Volume; US, Utility Score; VAS, Visual Analog Scale; VT, Vitality, energy, or fatigue

**Fig. 1:**
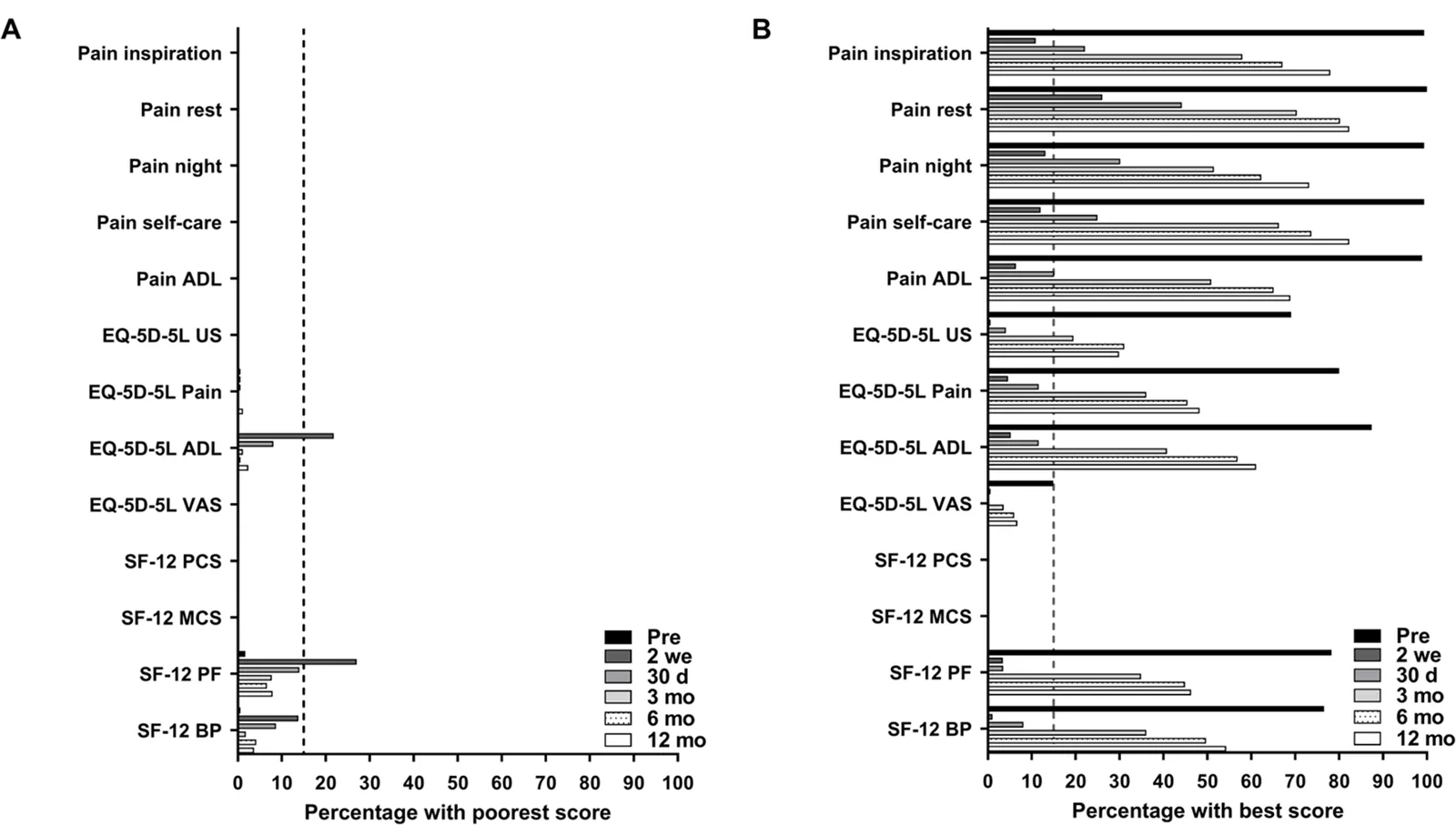
Floor (**A**) and ceiling (**B**) effects of the instruments at each follow-up visit in patients with multiple rib fractures. The dashed lines indicate 15% of patients as threshold for floor and ceiling effects. ADL, Activities of Daily Life; BP, Bodily Pain; d, days; EQ-5D, EuroQoL-5D Assessment with 5 Level response options; GH, General Health perceptions;; MCS, Mental Component Summary; MH, general Mental Health; mo, months PCS, Physical Component Summary; PF, Physical Functioning; pre, pre-trauma; QoL, Quality of Life; RE, Role limitations due to Emotional problems; RP, Role limitations due to Physical health; SF, Social Functioning; SF-12, Short Form-12; US, Utility Score; VAS, Visual Analog Scale; VT, Vitality, energy, or fatigue; we, weeks.

Floor effects were not present in most of the (sub)scales, except for the EQ-5D-5L ADL after 30 days and the SF-12 PF after 2 weeks (Fig. 1). Ceiling effects were present for all five pain questions from 30 days onwards, and for EQ-5D-5L utility score (US) from 3 months onwards. The EQ-5D-5L VAS and SF-12 PCS and MCS did not show a ceiling effect during follow-up.

### Minimal important change (MIC) and smallest detectable change (SDC)

Supplemental Table S2 shows the results of the anchor question. The data did not allow reliable assessment of the MIC and SDC, since correlation of the anchor question with the change scores did not exceed the minimally required correlation coefficient of 0.3 (data not shown).

## Discussion

Data from the current study showed that spirometry and pain during different activities are both valid instruments for monitoring functional recovery in patients after sustaining multiple rib fractures. Responsiveness was adequate for spirometry (FVC) and all pain items. The EQ-5D-5L and SF-12 are both valid, reliable, and responsive. Therefore, pain, HRQoL, and to a lesser degree also FVC are suitable instruments for measuring treatment effects over time.

This study is the first validation study on pain and pulmonary function as outcomes in patients suffering from multiple rib fractures. Validation studies in populations with different fracture types show similarities in expected PROM measurement properties after trauma. For example, Mahabier et al. showed ceiling effects for the DASH score at 12 months after humeral shaft fractures, where most patients fully recovered [29]. Increased rate of full recovery may also explain the ceiling effect for pain after 30 days and EQ-5D after 3 months in the current study.

Although a few recent rib fracture studies used PROMs, no validation was performed for these measurement instruments specifically in patients with rib fractures. Sundlof et al. analyzed pain and the relation to mental health in patients with ≥ 1 rib fractures (*n* = 72) using several Patient-Reported Outcomes Measurement Information System (PROMIS) questionnaires (v1.0 dyspnea characteristics, pain severity and work productivity and activity impairment questionnaire v2.0) [30]. Univariate analysis showed a significant association between patients with moderate to severe pain 2 weeks post injury and the presence of mental health symptoms. PROMIS questionnaires are validated tools for example in patients with lower extremity injuries [31] and upper extremity injuries [32], but validity for patients with rib fractures has not been confirmed.

As mentioned above, no PROM to date has undergone validation in a rib fracture population. The PAROS study (Pain After Rib Osteosynthesis) is ongoing and may produce results on the validity of BPI in due time [33]. Current data and Sundlof ‘s data present a good relationship between various questionnaires. A new HRQoL instrument designated for thoracic injuries might discover new information that general HRQoL instruments do not measure. Until such instrument becomes available and, the COSMIN systematic review (2019) recommends the use of EQ-5D-5L in combination with the Medical Research Council (MRC) dyspnea scale in rib fracture studies [12].

This study had limitations, the most important being that we were unable to determine MIC and SDC. Also, we used a handheld pulmonary function test in stead of incentive spirometry, as home visits were conducted for follow-up.

In conclusion, spirometry and pain during different activities are valid instruments for monitoring functional recovery in patients after sustaining multiple rib fractures. Responsiveness was confirmed for pain and spirometry (FVC). EQ-5D-5L and SF-12 are valid, reliable, and responsive. Pain, HRQoL, and to a lesser degree also FVC, are therefore suitable instruments for measuring treatment effects over time.

## Supplementary Information

Below is the link to the electronic supplementary material.

Supplementary Material 1

## Data Availability

No datasets were generated or analysed during the current study.

## Declarations

### Consortium members of the FixCon group

*The FixCon study group is composed of Taco J. Blokhuis MD PhD^2^, Doeke Boersma MD PhD^3^, Erik R. De Loos MD PhD^4^, Elvira R. Flikweert MD PhD^5^, Frank F.A. IJpma MD PhD^6^, Sanne Kleinveld MD PhD^7^, Simon P. Knops, MD PhD^8^, Albert F. Pull ter Gunne MD PhD^9^, W. Richard Spanjersberg MD^10^, Gerben Van der Bij MD PhD^11^, Floortje C. Van Eijck MD PhD^12^, Pieter J. Van Huijstee MD^13^, Gust Van Montfort MD^14^, Jefrey Vermeulen MD PhD^15^, and Dagmar I. Vos MD PhD^16^

^1^Trauma Research Unit Department of Surgery, Erasmus MC, University Medical Center Rotterdam, Rotterdam, The Netherlands

^2^Department of Surgery, Maastricht University Medical Center, Maastricht, The Netherlands

^3^Department of Surgery, Jeroen Bosch Ziekenhuis, ’s-Hertogenbosch, The Netherlands

^4^Department of Surgery, Zuyderland Medisch Centrum, Heerlen, The Netherlands

^5^Department of Surgery, Deventer Ziekenhuis, Deventer, The Netherlands

^6^Department of Surgery, University Medical Center Groningen, Groningen, The Netherlands

^7^Department of Surgery, Elisabeth-TweeSteden Ziekenhuis, Tilburg, The Netherlands

^8^Department of Surgery, Ikazia Ziekenhuis, Rotterdam, The Netherlands

^9^Department of Surgery, Rijnstate, Arnhem, The Netherlands

^10^Department of Surgery, Isala, Zwolle, The Netherlands

^11^Department of Surgery, Spaarne Gasthuis, Haarlem, The Netherlands

^12^Department of Surgery, Bravis Ziekenhuis, Roosendaal, The Netherlands

^13^Department of Surgery, HagaZiekenhuis, Den Haag, The Netherlands

^14^Department of Surgery, Catharina Ziekenhuis, Eindhoven, The Netherlands

^15^Department of Surgery, Maasstad Ziekenhuis, Rotterdam, The Netherlands

^16^Department of Surgery, Amphia Ziekenhuis, Breda, The Netherlands
