# Supplementary material for: Measurement properties of pulmonary function measurement and patient-reported outcome measures in patients with multiple rib fracture without flail segment; validation study alongside the multicenter FixCon trial
Source: Eur J Trauma Emerg Surg. 2026 Oct 9;52(1):315. doi: 10.1007/s00068-026-03338-7 (PMC13652763; doi:10.1007/s00068-026-03338-7)
Supplement: Supplementary file 1 [file 68_2026_3338_MOESM1_ESM.docx]

**Supplemental Figure 1: Pulmonary function at each follow-up visit in patients with multiple rib fractures**

Data are shown as box-whisker plots, with the boxes indicating the median and quartiles and the whiskers indicating the minimum and maximum scores.

d, days; FEV1, Forced Expiratory Volume in 1 second; FVC, Forced Vital Capacity; IC, Inspiratory Capacity; L, Liter; mo, months; TV, Tidal Volume; we, weeks.

**Supplemental Figure 2: Pain during inspiration (A), at rest (B), at night (C), and during self-care (D) and daily activities (E) at each follow-up visit in patients with multiple rib fractures**

Data are shown as box-whisker plots, with the boxes indicating the median and quartiles and the whiskers indicating the minimum and maxim scores.

ADL, activities of daily life; d, days; mo, months; we, weeks.

**Supplemental Figure 3: Short Form-12 (A and B) and EuroQoL-5D-5L (C and D) subscales at each follow-up visit in patients with multiple rib fractures**

Data are shown as box-whisker plots, with the boxes indicating the median and quartiles and the whiskers indicating the minimum and maxim scores. Dashed lines indicate the population-standardized values of 50 ± 10.

d, days; EQ-5D, EuroQoL-5D Assessment with 5 Level response options; MCS, Mental Component Summary; mo, months; PCS, Physical Component Summary; pre, pre-trauma; SF-12, Short Form-12; US, Utility Score; VAS, Visual Analog Scale; we, weeks.

**Supplemental Table 1: Predicted correlations between the instruments for (A) construct validity and (B) longitudinal validity in patients with multiple rib fractures**

**A. Construct validity**

| **(Sub)scale** | **Spirometry** | | | | | | **Pain** | | | | | **EQ-5D-5L** | | **SF-12** | |
| --- | --- | --- | --- | --- | --- | --- | --- | --- | --- | --- | --- | --- | --- | --- | --- |
|  | **TV (L)** | **FVC (L)** | **FVC (%)** | **IC (L)** | **FEV_1_ (L)** | **FEV_1_ (%)** | **Inspiration** | **Rest** | **Night** | **Self-care** | **ADL** | **US** | **VAS** | **PCS** | **MCS** |
| **Spirometry TV (L)** | 1 | Mod | **Mod** | Mod | Low | Low | Low | Low | Low | Low | Low | Low | Low | Low | Low |
| **FVC (L)** | Mod | 1 | High | High | High | **High** | Low | Low | Low | Low | Low | Low | Low | Low | Low |
| **FVC (%)** | **Mod** | High | 1 | **High** | **High** | High | Low | Low | Low | Low | Low | Low | Low | Low | Low |
| **IC (L)** | Mod | High | **High** | 1 | High | **High** | Low | Low | Low | Low | Low | Low | Low | Low | Low |
| **FEV_1_ (L)** | Low | High | **High** | High | 1 | High | Low | Low | Low | Low | Low | Low | Low | **Mod** | Low |
| **FEV_1_ (%)** | Low | **High** | High | **High** | High | 1 | Low | Low | Low | Low | Low | Low | Low | **Mod** | Low |
| **Pain Inspiration** | Low | Low | Low | Low | Low | Low | 1 | Hight | Mod | Mod | Mod | Low | Low | Low | Low |
| **Rest** | Low | Low | Low | Low | Low | Low | High | 1 | Mod | Mod | Mod | **Low** | Low | **Low** | Low |
| **Night** | Low | Low | Low | Low | Low | Low | Mod | Mod | 1 | Mod | Mod | Low | Low | Low | Low |
| **Self-care** | Low | Low | Low | Low | Low | Low | Mod | Mod | Mod | 1 | High | Mod | Low | Mod | Low |
| **ADL** | Low | Low | Low | Low | Low | Low | Mod | Mod | Mod | Hight | 1 | Mod | Low | **Mod** | Low |
| **EQ-5D-5L US** | Low | Low | Low | Low | Low | Low | Low | **Low** | Low | Low | Low | 1 | Mod | High | Mod |
| **VAS** | Low | Low | Low | Low | Low | Low | Low | Low | Low | Low | Low | Mod | 1 | Mod | Low |
| **EQ4 (pain)** | Low | Low | Low | Low | Low | Low | Mod | Mod | Mod | Mod | Mod | High | Mod | Mod | Low |
| **EQ3 (ADL)** | Low | Low | Low | Low | Low | Low | Low | Low | Low | Mod | **Mod** | High | Mod | **Mod** | Low |
| **SF-12 PCS** | Low | Low | Low | Low | **Mod** | **Mod** | Low | **Low** | Low | Low | **Low** | High | Mod | 1 | Low |
| **MCS** | Low | Low | Low | Low | Low | Low | Low | Low | Low | Low | Low | Mod | Low | Low | 1 |
| **PF** | Low | Mod | **Mod** | **Low** | Mod | **Mod** | Low | Low | Low | **Mod** | **Mod** | High | Mod | High | Low |
| **BP** | Low | Low | Low | Low | Low | Low | Low | **Low** | Low | Mod | Mod | Mod | Mod | High | Low |

**B. Longitudinal validity**

| **(Sub)scale** | **Spirometry** | | | | | | **Pain** | | | | | **EQ-5D-5L** | | **SF-12** | |
| --- | --- | --- | --- | --- | --- | --- | --- | --- | --- | --- | --- | --- | --- | --- | --- |
|  | **TV (L)** | **FVC (L)** | **FVC (%)** | **IC (L)** | **FEV_1_ (L)** | **FEV_1_ (%)** | **Inspiration** | **Rest** | **Night** | **Self-care** | **ADL** | **US** | **VAS** | **PCS** | **MCS** |
| **Spirometry TV(L)** | 1 | Low | Low | Low | Low | Low | Low | Low | Low | Low | Low | Low | Low | Low | Low |
| **FVC (L)** | Low | 1 | High | Mod | High | High | Low | Low | Low | Low | Low | Low | Low | Low | Low |
| **FVC (%)** | Low | High | 1 | Mod | High | High | Low | Low | Low | Low | Low | Low | Low | Low | Low |
| **IC (L)** | Low | Mod | Mod | 1 | **Mod** | High | Low | Low | Low | Low | Low | Low | Low | Low | Low |
| **FEV_1_ (L)** | Low | High | High | **Mod** | 1 | High | Low | Low | Low | Low | Low | Low | Low | Low | Low |
| **FEV_1_ (%)** | Low | High | High | High | High | 1 | Low | Low | Low | Low | Low | Low | Low | Low | Low |
| **Pain Inspiration** | Low | Low | Low | Low | Low | Low | 1 | High | Mod | Mod | Mod | Low | Low | Low | Low |
| **Rest** | Low | Low | Low | Low | Low | Low | High | 1 | Mod | **Mod** | Mod | Low | Low | Low | Low |
| **Night** | Low | Low | Low | Low | Low | Low | Mod | Mod | 1 | Mod | Mod | Low | Low | Low | Low |
| **Self-care** | Low | Low | Low | Low | Low | Low | Mod | Mod | Mod | 1 | High | Mod | Low | Mod | **Low** |
| **ADL** | Low | Low | Low | Low | Low | Low | Mod | **Mod** | Mod | High | 1 | Mod | Low | Mod | Low |
| **EQ-5D-5L US** | Low | Low | Low | Low | Low | Low | Low | Low | Low | Mod | Mod | 1 | **Mod** | **Mod** | Mod |
| **VAS** | Low | Low | Low | Low | Low | Low | Low | Low | Low | Low | Low | Mod | 1 | Mod | Low |
| **EQ4 (pain)** | Low | Low | Low | Low | Low | Low | Low | Low | Low | **Mod** | Mod | **High** | **Mod** | Mod | Low |
| **EQ3 (ADL)** | Low | Low | Low | Low | Low | Low | Low | Low | Low | Low | **Mod** | High | Mod | Mod | Low |
| **SF-12 PCS** | Low | Low | Low | Low | Low | Low | Low | Low | Low | **Mod** | Mod | Mod | Mod | 1 | Low |
| **MCS** | Low | Low | Low | Low | Low | Low | Low | Low | Low | Low | Low | **Mod** | Low | Low | 1 |
| **PF** | Low | Low | Low | Low | Low | Low | Low | Low | Low | **Mod** | **Mod** | Mod | **Mod** | High | Low |
| **BP** | Low | Low | Low | Low | Low | Low | Low | Low | Low | Mod | Mod | Mod | **Mod** | High | Low |

Expected strength of correlation for all possible combinations; r>0.6 indicates high correlation, 0.3<r>0.6 moderate correlation, and r> 0.6 low correlation. The correlations given above the self-correlation are also mentioned in the columns to the left as reversed correlation and are thus superfluous.

ADL, Activities of Daily Life; BP, Bodily Pain; EQ-5D, EuroQoL-5D Assessment with 5 Level response options; FEV1, Forced Expiratory Volume in 1 second; FVC, Forced Vital Capacity; IC, Inspiratory Capacity; L, Liter; MCS, Mental Component Summary; PCS, Physical Component Summary; PF, Physical Functioning; QoL, Quality of Life; SF-12, Short Form-12; TV, Tidal Volume; US, Utility Score; VAS, Visual Analog Scale.

**Supplemental Table 2: Answers to the transition item at four different time windows in patients with multiple rib fractures**

| **Transition item** |  | **Time window** | | | |
| --- | --- | --- | --- | --- | --- |
|  | **Total**  **(N=168)** | **14 to 30 days**  **(N=0)** | **30 days to 3 months**  **(N=40)** | **3 to 6 months**  **(N=67)** | **6 to 12 months**  **(N=61)** |
| **Pulmonary function** |  |  |  |  |  |
| 1 ‘much better’ | 33 (20%) | N.A. | 11 (28%) | 12 (18%) | 10 (16%) |
| 2 ‘slightly better’ | 49 (29%) | N.A. | 16 (40%) | 17 (25%) | 16 (26%) |
| 3 ’no change’ | 77 (46%) | N.A. | 13 (33%) | 33 (49%) | 31 (51%) |
| 4 ‘slightly worse’ | 9 (5%) | N.A. | 0 (0%) | 5 (7%) | 4 (7%) |
| 5 ‘much worse’ | 0 (0%) | N.A. | 0 (0%) | 0 (0%) | 0 (0%) |
|  |  |  |  |  |  |
|  | **Total**  **(N=208)** | **14 to 30 days**  **(N=38)** | **30 days to 3 months**  **(N=41)** | **3 to 6 months**  **(N=68)** | **6 to 12 months**  **(N=61)** |
| **General complaints** |  |  |  |  |  |
| 1 ‘much better’ | 85 (41%) | 16 (42%) | 25 (61%) | 27 (40%) | 17 (28%) |
| 2 ‘slightly better’ | 58 (28%) | 14 (37%) | 13 (32%) | 16 (24%) | 15 (25%) |
| 3 ’no change’ | 58 (28%) | 5 (13%) | 3 (7%) | 24 (35%) | 26 (43%) |
| 4 ‘slightly worse’ | 5 (2%) | 3 (8%) | 0 (0%) | 0 (0%) | 2 (3%) |
| 5 ‘much worse’ | 2 (1%) | 0 (0%) | 0 (0%) | 1 (1%) | 1 (2%) |

Data are shown as number with valid percentage.

N.A., not applicable.
